# Accidental Detection of Cocaine in Urine in Pediatric Patients: Case Series and Literature Review

**DOI:** 10.3390/children11111301

**Published:** 2024-10-28

**Authors:** Martina Focardi, Ilenia Bianchi, Marta Romanelli, Valentina Gori, Laura Nanni, Fabio Vaiano, Stefania Losi

**Affiliations:** 1 Forensic Pathology Unit, AOU Careggi, Largo Brambilla 3, 50134 Florence, Italy; martina.focardi@unifi.it (M.F.); marta.romanelli@unifi.it (M.R.); valentina.gori@unifi.it (V.G.); 2 Laboratory of Personal Identification and Forensic Morphology, Department of Health Sciences, University of Florence, Largo Brambilla 3, 50134 Florence, Italy; 3 Pediatric Emergency Unit, Department of Intensive Care and Emergency, Meyer Children’s Hospital IRCCS, Viale Pieraccini 24, 50139 Florence, Italy; laura.nanni@meyer.it; 4 FT-LAB Forensic Toxicology Laboratory, Department of Health Science, University of Florence, Largo Brambilla 3, 50134 Florence, Italy; fabio.vaiano@unifi.it; 5 Responsible GAIA Service, Meyer Children’s Hospital IRCCS, Viale Pieraccini 24, 50139 Florence, Italy; stefania.losi@meyer.it

**Keywords:** child abuse, maltreatment, drug exposure, forensic toxicology, toxicological analyses, substance of abuse, cocaine, pediatric health

## Abstract

Infantile occult exposure to cocaine in domestic environments represents a complex clinical and medico-legal problem, which can be associated with abuse and neglect and with potential short- and long-term health risks for children. The authors present a retrospective study on 764 children under 14 years old who accessed the Emergency Department of IRCCS Meyer from 2016 to 2023 and were included in the GAIA (Child and Adolescent Abuse Group) protocol for suspected maltreatment and abuse, and for which a urine toxicology analysis was performed. The aim is to discuss the medico-legal implications and highlight the need for a thorough evaluation and management of such situations. Urine screening tests for substances of abuse (e.g., cocaine, opiates, etc.) were performed with an EMIT^®^ Siemens VIVA-E drug testing system (Siemens, Newark DE) in 124 cases for which the child’s clinical condition raised suspicion of intoxication, or the family context indicated distress or substance abuse dependency. The screening results revealed the presence of cocaine and its main metabolite, benzoylecgonine, in the urine of 11 children. In one case, a single girl was brought to the Emergency Department by staff from the facility where she and her mother were staying. In most of the cases, children were brought to the Emergency Department by their parents who accessed the Emergency Department due to various clinical manifestations (drowsiness, agitation, seizures, hypotonia, diarrhea, vomiting, etc.), except for one case of eye trauma suspected to be caused by abuse or neglect by one of the parents. Three of the children did not have signs or symptoms attributable to substance exposure, whilst eight of the cases presented some of the symptoms associated with occult infant exposure to cocaine, such as neurological manifestations, seizures, gastrointestinal symptoms, and respiratory depression. The probable mode of intake was mostly through breastfeeding and continuous environmental exposure due to domestic contamination or inhalation of “crack”. In the case of a 12-hour-old infant, there was probable prenatal in utero exposure. All the children were hospitalized, some for medical reasons and others solely as a precautionary measure for proper care. In all cases, a report was made to the Prosecutors as required by the Italian Penal Code, as well as to the Court of Minor. The study highlighted the importance of a multidisciplinary approach involving pediatricians, social workers, and forensics, as well as close collaboration with the relevant authorities, as the Gaia service at IRCCS Meyer offers. The occasional detection of cocaine in cases that showed no suspicion of intoxication led to a modification of the procedure and the development of a standardized protocol at IRCCS Meyer both in terms of prevention and in the detection and interception of hidden cases, in order to intervene early and initiate the necessary care pathways (secondary prevention). This protocol includes routine toxicological urine testing in all suspected or confirmed cases of child abuse, not just in those where symptoms might suggest a suspicion of intoxication.

## 1. Introduction

Infantile occult exposure to cocaine in the home environment represents a complex clinical and medico-legal problem, which can be the basis of situations of abuse and neglect, with potential short- and long-term health risks for children. The accidental detection of cocaine in the urine of children in cases of suspected maltreatment is a known occurrence. Numerous studies, particularly American ones, have highlighted this problem, especially with crack inhalation, with very extensive case series. As stated by the United Nations Convention on the Rights of the Child (UNCRC) of 1989 [1], it is important to safeguard children from the widespread illicit use of narcotic drugs and psychoactive substances, a well-known phenomenon.

Exposure can happen through several avenues, including intrauterine, lactation, intentional feeding, accidental (unintentional) ingestion of cocaine, or domestic contamination from cocaine dust through hand–mouth activity and the passive breathing in of crack fumes when freebase cocaine is smoked by adults who care for them [2,3]. Drug tests are frequently conducted in situations involving unexplained irritability or changes in the consciousness of an infant or children, but they may be overlooked when non-specific symptoms are presented. Given the significant overlap, drug exposure should be assessed alongside other forms of child maltreatment, such as physical abuse [4,5]. The detection of drug exposure in a child can have crucial implications for their safety and the planning of their care [5].

The authors present a case series of accidental detection of cocaine in children’s urine to discuss the medico-legal implications and highlight the need for a thorough evaluation and management of such situations.

## 2. Materials and Methods

This retrospective study analyzed the records of the Emergency Department of IRCCS Meyer from 2016 to 2023 for the maltreatment and abuse of subjects under 14 years old (thus excluding those cases where cocaine positivity could be due to recreational use in adolescents) included in the GAIA (Child and Adolescent Abuse Group) pathway, for which a urine toxicology analysis was performed. The Gaia service at IRCCS Meyer is a service dedicated to the protection of children’s rights, composed of a multidisciplinary team of specialists (pediatricians, psychologists, child neuropsychiatrists, nurses, and social workers) with the possibility of consulting a forensic expert of Careggi Hospital of Florence.

The variables analyzed were age, sex, social context of cohabitation, reason for hospital admission, possible source of intake, and healthcare actions for the child’s protection. Only cases with positive urine cocaine tests were considered.

Urine samples were tested using an EMIT^®^ Siemens VIVA-E drug testing system (Siemens, Newark, DE, USA) to examine commonly abused substances including cocaine, opiates, cannabinoids, amphetamines, barbiturates, methadone, and benzodiazepines, following the manufacturer’s guidelines.

## 3. Results

The study was completed on a total of 764 children who accessed the Emergency Department of IRCCS Meyer from 2016 to 2023 and were included in the GAIA protocol for suspected maltreatment and abuse. Urine screening tests for substances of abuse were performed in 124 cases involving children under the age of 14 for the following reasons: first of all, the child’s clinical condition raised suspicion of intoxication, and on the second hand, the family context indicated distress or substance abuse dependency.

**Figure 1:**
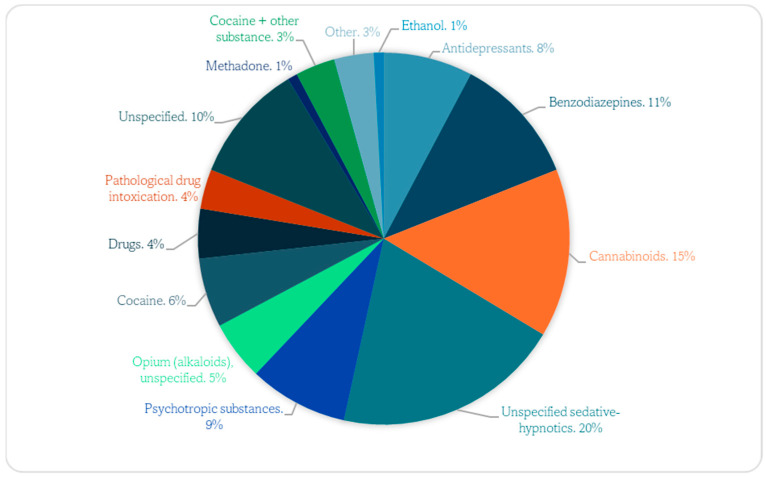
Substances of abuse distribution in the sample.

The screening results detected the presence of cocaine and its principal metabolite, benzoylecgonine, in the urine of 11 children. There was a higher frequency of positivity for other xenobiotics (cannabinoids/benzodiazepines) (Figure 1).

In Table 1, the cases are illustrated. With reference to age, it ranges from 2 months to 34 months, except for one subject who was approximately 12 h old. There were four females and seven males. In one case, a single girl was brought to the Emergency Department by staff from the facility where she and her mother were staying. In another case, the report was made after the child was found next to his mother, who was homeless and near death. In the remaining cases, the children were brought to the Emergency Department by their parents. The reasons for access to the Emergency Department are mostly due to various clinical manifestations (drowsiness, agitation, seizures, hypotonia, diarrhea, vomiting, etc.), except for one case of eye trauma suspected to be caused by abuse or neglect by one of the parents; three of the children did not have signs or symptoms attributable to substance exposure; and eight of the cases presented some of the symptoms associated with occult infant exposure to cocaine, such as neurological manifestations, seizures, gastrointestinal symptoms, and respiratory depression.

The probable mode of intake was mostly through breastfeeding and continuous environmental exposure due to domestic contamination or inhalation of “crack”. In one case concerning a 12-hour-old infant, there was probable prenatal in utero exposure. In all cases, a report was made to the Prosecutors as required by the Italian Penal Code, as well as to the Court of Minor for situations of risk to the child attributable to the behaviors of parents or family members to whom the child was entrusted. All the children were hospitalized, some for medical reasons and others solely as a precautionary measure for proper care.

## 4. Discussion

Exposure to drugs of abuse during prenatal development and childhood is an emerging public health issue, potentially resulting in numerous adverse effects on children’s health, often associated with abuse and neglect [3,6]. According to the Italy Country Drug Report 2019 [7], substance use among young adults (aged 15–34) in Italy shows a significant prevalence of cannabis (20.9%), followed by cocaine (1.7%), MDMA (0.8%), and amphetamines (0.3%). Children at risk of drug exposure, according to the National Alliance for Drug Endangered Children, are those who may experience physical or emotional harm due to the use, possession, production, cultivation, or distribution of substances by their caregivers. It may also be parents whose drug abuse interferes with their parenting skills and therefore cannot provide a secure environment for the child. In addition, 1 in 35 children live in a family with at least one parent suffering from an illicit drug use disorder [8]. Approximately 2.9 million children under the age of 6 live with at least one parent affected by substance use disorder [9]. Since there is an association between parental use of drugs and child abuse, drug exposure is acknowledged as an important aspect in assessing children in situations of suspected abuse or neglect [5,10]. Identifying drug exposure in a child can have significant consequences for safety and influence child welfare protection strategies [5].

As stated by the United Nations Convention [1] on the Rights of the Child (UNCRC) of 1989, it is crucial to safeguard children from illegal drug use and psychoactive substances, a widespread and well-known phenomenon. The accidental detection of cocaine in the urine of children in instances of alleged maltreatment is a known phenomenon. Numerous studies have highlighted this issue, especially in the United States, where the inhalation of crack cocaine is common, with even very large case series. Several studies [11,12,13] report cases of cocaine-positive urine in children, where the probable mechanism of exposure is the passive inhalation of vapors when freebase cocaine is smoked by adults who care for them. This finding aligns with our own case series, where one of the likely modes of exposure was indeed this. Rivkin and Gilmore describe a case of cocaine detection in the urine of a child following the unintentional ingestion of cocaine in a domestic setting, which resulted in generalized tonic–clonic seizures [14]. The study by Sigmund J. Kharasch [2] identified six cases of cocaine positivity over a six-week period among 250 urine samples from children, despite the absence of clinical manifestations. Petska’s case series shows 15 cases of cocaine-positive urine from 2013 to 2017, of which 4 presented neurological symptoms (apnea, loss of consciousness, seizures, lethargy), 1 presented vomiting and agitation, and the remaining cases showed no symptoms [5]. Many earlier studies on substance exposure in abused children have been restricted by the inclusion of teenagers (with potential recreational use) [5]. There are various possible routes of cocaine exposure. One possible route is prenatal, in utero. The placenta is essential for transferring nutrients and drugs from the mother to the fetus, and for removing waste products from fetal metabolism. Significant levels of cocaine have been found in amniotic fluid, potentially leading to extended fetal exposure to the drug [3,15]. This study identified a single case of cocaine positivity in a newborn just 12 h old, likely due to prenatal in utero exposure, consistent with previous studies [13,16] showing that cocaine can be detected in the urine of newborns up to 3–5 days after birth if in utero exposure occurred. After birth, potential routes of cocaine exposure include breastfeeding, as substances are excreted in breast milk, intentional administration, accidental (unintentional) ingestion of cocaine, or domestic contamination by cocaine dust through normal hand-to-mouth activity, and passive inhalation of “crack” vapors when freebase cocaine is smoked by adults who care for them [2,3,17]. In line with the above, in our case series, the likely modes of exposure were mostly breastfeeding and continuous environmental exposure through domestic contamination or “crack” inhalation. In our study, eight cases presented with neurological manifestations, including generalized hypotonia, the inability to maintain a sitting position, seizures, gastrointestinal symptoms, and respiratory depression, consistent with the symptoms and signs of intoxication described in previous studies [5,10]. Although the results of our study did not identify upper and lower respiratory tract symptoms as clinical manifestations associated with cocaine exposure, it is still useful to pay attention to these symptoms in cases of suspected abuse, as Lustbader et al. [13] significantly correlates them with positive urine results. In this study, 3 of the 11 children who tested positive for cocaine had no signs or symptoms attributable to substance exposure. The incidental finding of cocaine in urine, even in cases without any suspicion of intoxication, is consistent with previous studies showing a significant number of children seeking hospital care for unrelated issues despite having been exposed to cocaine [5,13,18]. Regarding the immediate and long-term effects associated with cocaine exposure in infants and children, the correlation between prenatal cocaine exposure and the development of long-term complications has been studied. Cocaine use in pregnancy is correlated with increasing complications (from migraine and seizures to premature membrane rupture, hypertensive crises, spontaneous abortion, preterm labor, and complicated delivery). Fetal cocaine exposure is linked to an enhanced risk of preterm delivery and evidence at birth of low weight, decreased head circumference, and length compared to infants not exposed to cocaine during pregnancy. Additionally, exposure to this substance during fetal development is associated with the later onset [19] of behavioral and learning disorders [20,21], as well as language and memory impairments [22].

Regarding postnatal cocaine exposure, there are few studies examining the impacts of cocaine exposure during infancy and the infant outcomes of cocaine use during breastfeeding. Some studies have found an association between active or passive cocaine exposure during infancy (in children aged 8 years or younger) and the onset of complications immediately following substance intake. Some authors have noted that if substance intake occurs during breastfeeding, the cocaine in breast milk is orally absorbed to a limited extent by the breastfed infant, as it is generally broken down in the intestine, potentially exposing the infant to limited amounts of the substance. However, given that the ability to metabolize and eliminate drugs like cocaine is not fully developed in infants, exposure even to small doses of cocaine could persist and cause significant harm [23].

Neurological complications (focal seizures) have been reported without signs of structural brain damage [24], as well as cardiovascular complications (tachycardia) [25]. Other symptoms correlated with the substance dose include an altered/depressed mental state, agitation, delirium, gastrointestinal symptoms, fever, hypertension, respiratory depression, cyanosis, mydriasis, ataxia, and dizziness. Regarding long-term damage, cocaine exposure in infancy could lead to attention or memory deficits and behavioral changes. Other authors also emphasize how substance exposure through breastfeeding can cause both short- and long-term effects in infants [26].

Therefore, the published experience on cocaine exposure in neonates and children in the postnatal period is limited. Specifically, the literature contains studies that have mostly analyzed the short-term complications of cocaine exposure during childhood, with the correlation between childhood cocaine exposure and the development of long-term complications remaining under-researched. This concept has significant medico-legal implications for understanding potential long-term effects and determining whether these could constitute personal injury based on the duration of the illness.

Drug exposure can be examined through hair and urine testing. Hair testing is extensively utilized in forensic sciences because of its benefits, including non-invasive collection, an extended detection period, and straightforward sample preservation [27]. However, adult cut-offs cannot be used for children, as their hair is more porous and grows at varying rates [28], making it difficult to interpret [3].

Thus, hair tests can identify past exposures but may be difficult to interpret [29] compared to urine toxicology tests, which provide more immediate, accurate information with a detection window for cocaine of just a few days. In line with the above, for all cases of children who tested positive for cocaine, a hair toxicology analysis was ordered to assess a broader detection period.

Regarding the legal framework on the subject of child maltreatment, criminal law defines the crime as “maltreatment against family members and cohabitants”, and the cases under consideration can fall within the criteria of this crime, but other criminal offenses committed against minors must be considered (violation of family assistance obligations or personal injury).

The different forms of child maltreatment assume varying relevance from a forensic perspective, but they are all offenses that can be prosecuted ex officio, for which healthcare professionals have a mandatory obligation to report to the Judicial Authority. The knowledge of this obligation is essential for professionals because it is through the report that the protective measures for the minor, as provided by our legal system, can be initiated. There is direct protection, provided by the civil and administrative system and implemented through the measures of the Juvenile Court and the interventions of Social Services, in all cases of “harm” to the minor attributable to the behavior of the parents or family members responsible for the child, regardless of any criminal implications.

In the specific case, regardless of the crime involved, in all instances where children tested positive for cocaine, reports were made both to the judicial authorities (Prosecutor) and the Juvenile Court in situations where there was “harm” to the minor attributable to the behavior of the parents or family members responsible for the child.

The strengths of the study include the use of an extensive urine drug test that detects recent contact with both illicit and pharmaceutical substances, and unlike many previous studies, cases where cocaine positivity might be due to recreational use in adolescents were excluded, including only subjects under 14 years of age in the study. Additionally, by including neonates under 4 days old in the sample, we considered potential positive urine samples caused by maternal exposure to cocaine during pregnancy.

### Study Limitations

A significant limitation of the study is related to the sample size and the fact that, being a retrospective study, not all variables necessary for a complete understanding of the phenomenon were collected.

## 5. Conclusions

The study has highlighted how a multidisciplinary approach is essential, involving pediatricians, social workers, and forensic doctors, as well as close collaboration with the relevant authorities. In fact, the Gaia service at IRCCS Meyer is a service dedicated to the protection of children’s rights, composed of a multidisciplinary team of specialists (pediatricians, psychologists, child neuropsychiatrists, nurses, and social workers) who, through attentive listening, are able to identify potential risk factors and intervene promptly with a preventive approach.

The occasional detection of cocaine in cases that showed no suspicion of intoxication led to a modification of the procedure and the development of a standardized protocol at IRCCS Meyer. This protocol includes routine toxicological urine testing in all suspected or confirmed cases of child abuse, not just in those where symptoms might suggest a suspicion of intoxication.

The results of this study show how fundamentally important is the Gaia protocol, both in terms of prevention and in the detection and interception of hidden cases, in order to intervene early and initiate the necessary care pathways (secondary prevention).

## Figures and Tables

**Table 1:** Sample description: 11 cases considered.

Age/Sex	Reason for Access	Physical Examination	Urine Toxicology
12 months and 12 days/Female	Irritability and agitation. In medical history: facial trauma at age 10 months due to accidental fall (apparently neglect by her father).	Negative. Abrasions on the bridge of the nose (fall from bed).	Positive for COCAINE
14 months and 21 days/Male	Hyporeactivity and hypothermia following vomiting; vomiting started from the same evening, profuse, 10 episodes in 3 h, one discharge of diarrhea	Child dejected, whining, cold extremities, isochoric pupils normoreactive to light, not nystagmus	Positive for COCAINE
2 years and 10 months/Female	Right eye trauma	Alert, responsive, normal tone, right periocular bruise, crusty lesion on lip, pupils equal and reactive to light	Positive for COCAINE
12 h/Male	EEG at 25 weeks, urgent CT scan, BCF alterations	Transferred to Meyer Neonatal Intensive Care Unit for severe asphyxia and respiratory distress	Positive for COCAINE + alcohol
14 months/Female	Inability of the child to maintain a sitting position since the previous day	Generalized hypotonia, irritability, inability to maintain sitting position; negative cranial CT scan	Positive for COCAINE + benzodiazepines
2 months/Female	Apnea episode following milk regurgitation for suspected GERD (breastfeeding)	Negative	Positive for COCAINE
15 months/Male	Subsequent tonic–clonic seizures with perioral cyanosis lasting 1 min (treated with Micropam); transferred to Meyer Hospital at the 3rd episode	Negative; neurological symptoms were not compatible with the negativity of the EEG so other causes were investigated (urine toxicology)	Positive for COCAINE
17 months/Male	Generalized hypotonia	Generalized hypotonia	Positive for COCAINE and cannabinoids
3 months and 9 days/Male	Found next to his mother who died of an overdose	Respiratory depression	Positive for COCAINE and ethyl glucuronide
1 year and 7 months/Male	Irritability and agitation	Vigilant, normoreactive isochoric pupils	Positive for COCAINE
2 years and 9 months/Male	Brother showed intoxication symptoms	Negative	Positive for COCAINE

## Data Availability

The original contributions presented in the study are included in the article, further inquiries can be directed to the corresponding author.
